# Efficacy of Osimertinib in NSCLC Harboring Uncommon EGFR L861Q and Concurrent Mutations: Case Report and Literature Review

**DOI:** 10.3389/fonc.2021.731572

**Published:** 2021-09-02

**Authors:** Ruiting Lin, Ruilian Chen, Zhiqiang Chen, Leihao Hu, Wei Guo, Zexin Zhang, Lizhu Lin, Hanrui Chen

**Affiliations:** ^1^First Clinical Medical College, Guangzhou University of Traditional Chinese Medicine, Guangzhou, China; ^2^Department of Oncology, The First Affiliated Hospital of Guangzhou University of Traditional Chinese Medicine, Guangzhou, China

**Keywords:** epidermal growth factor receptor, non-small-cell lung cancer, osimertinib, compound mutation, tyrosine kinase inhibitor, uncommon mutation, L861Q mutation

## Abstract

The efficacy of first-and second-generation epidermal growth factor receptor tyrosine kinase inhibitors (EGFR-TKIs) in NSCLC patients with the EGFR L861Q mutation has been studied previously. However, there is little evidence on the efficacy of osimertinib in NSCLC patients with uncommon mutations. Here, we report the case of a 68-year-old man with advanced NSCLC with concurrent EGFR L861Q mutation as well as TP53 and RB1 mutations. The patient was treated with osimertinib as first-line therapy and achieved a remarkable progression-free survival of 15 months. His symptoms were significantly alleviated and the dose was well tolerated. The findings of the present study indicate that osimertinib might be a good treatment option for NSCLC patients with the L861Q mutation.

## Introduction

Epidermal growth factor receptor tyrosine kinase inhibitors (EGFR-TKIs) have revolutionized the therapeutic paradigm for advanced non-small cell lung cancer (NSCLC) with EGFR mutations. Common EGFR mutations account for approximately 75% to 80% of EGFR-mutant NSCLC cases, including exon 19 deletion and the L858R mutation, which has been reported to improve the efficacy of EGFR-TKIs in clinical trials. The remaining EGFR-mutant cases are a highly heterogeneous group of genomic alterations within EGFR exons 18–21 (1, 2). Currently, with the widespread use of next-generation sequencing (NGS), an increasing number of rare or atypical EGFR mutations have been identified. However, further insights are required on the efficacy of EGFR-TKIs against advanced NSCLC harboring uncommon EGFR mutations, especially the efficacy of third-generation EGFR-TKIs such as osimertinib. Here, we present the case of a patient harboring the L861Q mutation, who maintained a sustained response to osimertinib. Written informed consent was provided by the patient to use case details and the accompanying images for publication.

## Case Report

**Figure 1:**
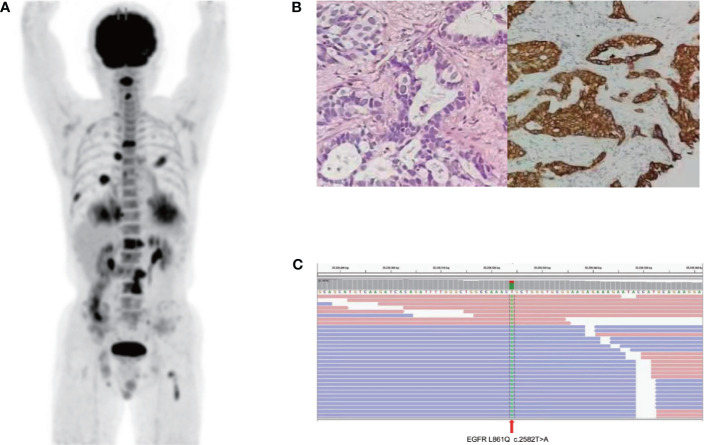
Baseline data: **(A)** Positron emission tomography-computed tomography (PET-CT) image. **(B)** Representative histopathological image of the tumor (H&E staining). **(C)** Next-generation sequencing showed an L861Q mutation in the epidermal factor receptor exon 21.

**Figure 2:**
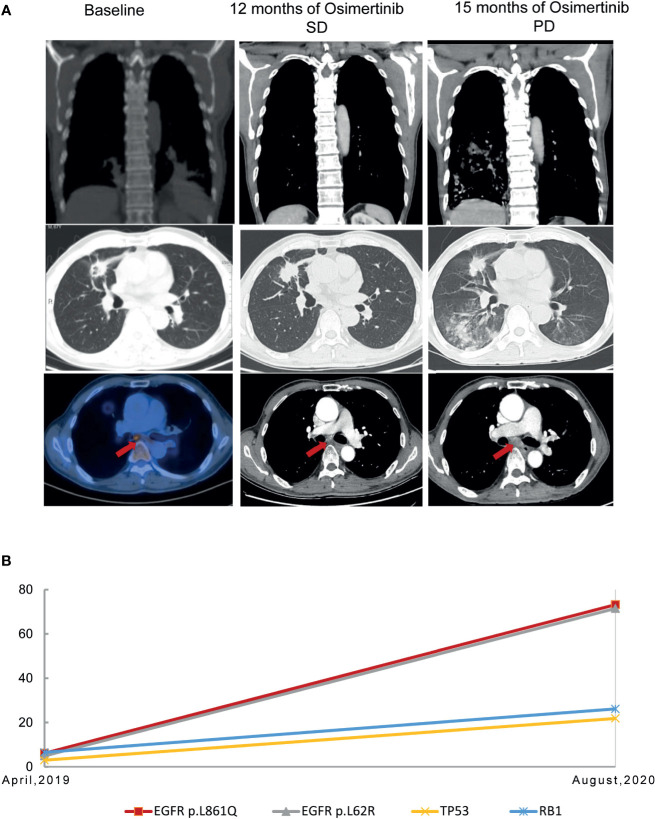
Representative computed tomography images at various points **(A)** and mutation analysis of the patient’s plasma **(B)** before and after osimertinib treatment.

A 68-year-old, nonsmoker, male presented with a history of back pain for two months; no family history of tumor was reported. Positron emission tomography-computed tomography (PET-CT) revealed a fluoro-2-deoxy-d-glucose (FDG)-positive lesion in the left middle lung lobe and metastases in multiple bones (**Figure 1A**). CT-guided core needle biopsy of the tumor revealed adenocarcinoma with positivity for CK7 protein and TTF-1 staining (**Figure 1B**). To identify potentially actionable mutations of the patient, paired NGS‐based genetic testing of 1,021 cancer‐related genes was performed with both circulating free DNA from plasma and DNA extracted from the leukocytes (Geneplus‐Beijing Ltd., Beijing, China). EGFR L861Q mutations (allelic fraction, AF=6.1%) in exon 21 were identified by next-generation sequencing (NGS) of the plasma, with concurrent TP53 N239S mutation in exon 7 and RB1 mutations (**Figure 1C**). First-line therapy with osimertinib (80 mg daily) was initiated. He achieved stable disease condition with decreasing primary lesions, confirmed based on the Response Evaluation Criteria in Solid Tumors 1.1. The patient also showed significant improvement in terms of back pain and quality of life, and the adverse events were well tolerated. He experienced progressive disease of the right frontal lung lobe, subcarinal lymph node, and brain metastases after a progression-free survival (PFS) of 15.0 months (**Figure 2A**). Subsequently, the patient presented with severe cough, headache, and back pain.

**Figure 3:**
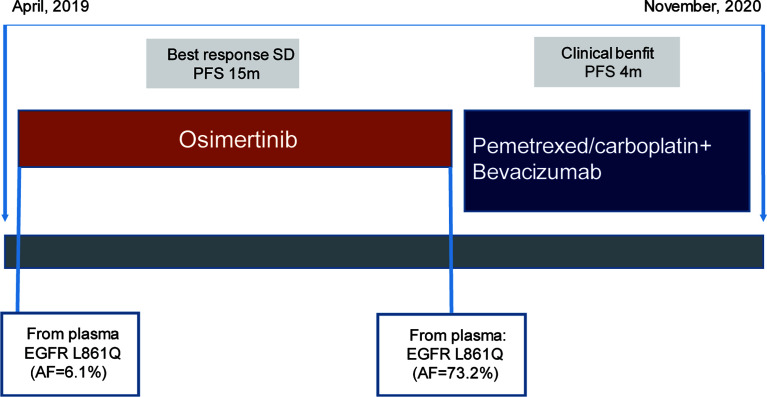
Schematic of the course of disease management, showing different treatment regimens prescribed and the results of the mutation analyses.

Due to the infeasibility of obtaining additional tissue biopsy, liquid biopsy assessing circulating tumor DNA (ctDNA) by NGS was performed. The AF of the L861Q mutation increased to 73.2%, with TP53 and RB1 mutations and absence of EGFR T790M (**Figure 2B**). Subsequently, the patient was treated with pemetrexed and carboplatin plus bevacizumab as second-line therapy. After two cycles of chemotherapy, he experienced significant improvement in headache, cough, and back pain, but experienced fatigue. However, the patient refused to continue chemotherapy because of personal reasons. The last follow-up was in November 2020, after which the patient passed away, with an overall survival of 19 months (**Figure 3**).

## Discussion

Here, we describe the clinical efficacy of first-line osimertinib in patients with advanced NSCLC harboring concurrent uncommon EGFR, TP53, and RB1 mutations. The patient benefitted from treatment with osimertinib, with a PFS of 15 months. To the best of our knowledge, this is the first report on the clinical effects of first-line osimertinib in Chinese NSCLC patients carrying the concurrent EGFR L861Q mutation as well as TP53 and RB1 mutations.

Owing to the high heterogeneity and low prevalence of NSCLC with rare EGFR mutations, there is no prospective clinical trial data that directly compare different EGFR TKIs, or chemotherapy in advanced patients with uncommon EGFR mutations. Thus, optimal first-line therapy is still undetermined. The L861Q mutation is the second most common mutation among rare EGFR mutations. Previous studies show inconsistent results regarding the efficacy of first-generation EGFR-TKIs against advanced NSCLC with uncommon EGFR mutations. Preclinical research shows that NSCLC harboring EGFR L861Q is resistant to gefitinib or erlotinib and might be sensitive to afatinib or osimertinib (3–6). Floc’h et al. study reported osimertinib inhibited signaling pathways and cellular growth in cell lines or patient-derived xenografts harboring uncommon EGFR mutations (6). Clinical evidence on efficacy of first-generation EGFR-TKIs in patients with uncommon EGFR mutations is variable. In the NEJ002 trial, the objective response rate (ORR) and PFS of gefitinib were 20% and 2.2 months in NSCLC patients harboring uncommon EGFR mutation (G719X or L861Q), respectively, which were significantly lower than that of 76% and 11.4 months in those with common EGFR mutations (7). Several retrospective studies reported that the ORR was 40-60% and the PFS was 5.2-8.9 months following treatment with first-generation EGFR TKIs in NSCLC patients harboring L861Q mutations (8–11). A *post hoc* analysis of the LUX-Lung 2, LUX-Lung 3, and LUX-Lung 6 trials showed that patients harboring the L861Q mutation had an ORR of 56%, median PFS of 8.2 months, and OS of 17.1 months when treated with afatinib (12). On the basis of these findings, afatinib has been approved by the United States Food and Drug Administration and the European Medicine Agency for the first-line therapy in NSCLC patients with EGFR uncommon mutations. A phase II clinical study reported that nine advanced NSCLC patients harboring EGFR L861Q mutation who received osimertinib therapy achieved median PFS of 15.2 months and an ORR of 78% (13). Similarly, our present Chinese NSCLC patient harboring EGFR L861Q mutations also had a PFS of 15 months with osimertinib treatment. Sensitizing EGFR-mutant patients obtained the PFS of 18.9 months and an ORR of 80% for osimertinib in the FLAURA trail (14). The benefit of osimertinib for patients sensitive EGFR mutation seemed to be superior than those with EGFR L861Q mutation. Passaro et al. have showed the recent advances on the role of EGFR-TKI in patients with uncommon EGFR mutations and considered afatinib or osimertinib as possible first-line treatment options for major uncommon EGFR mutations (15).

An important implication of the present case study is the importance of NGS and liquid biopsy in detecting alterations in molecular abundance. Dynamic genetic changes can occur in lung cancers. Biopsy tissue can only provide limited information owing to heterogeneity of the tumor. Furthermore, performing a biopsy is relatively complicated, with some parts being difficult to access, and repeated sampling causes great pain to patients. A previous study showed that dynamic changes in mutation abundance can reflect the efficacy of EGFR-TKIs and that a rapid decrease in mutation abundance predicts a better EGFR-TKI response (16, 17). Thus, dynamic monitoring of gene aberrances in ctDNA and generation of an integrated genomic profile from NGS can help tailor targeted treatment options for patients.

EGFR-mutant NSCLC patients with TP53 mutations showed inferior response and poor prognosis for EGFR-TKI, especially those with exon 6-8 mutation (18, 19). Besides, different categories of the TP53 status have been reported as a prognostic marker for patients with EGFR-TKI therapy (20). TP53 exon 8 mutations demonstrated a role in inferior clinical outcome in patients with the first and second generations of EGFR-TKI, which also confirmed the negative impact in patients with osimertinib (21). A phase III clinical trial evaluating the use of osimertinib in untreated advanced NSCLC patients with concurrent EGFR and TP53 mutations has been registered on the ClinicalTrials.gov website (Identifier: NCT04695925). Concurrent mutations in EGFR-mutant lung cancers may contribute to tumor heterogeneous outcomes and associate with resistance to EGFR-TKI treatment (22, 23). A study showed that concurrent RB1 and TP53 alterations in EGFR-mutant patients were at unique risk of histologic transformation and inferior response (24). The patient in our case harbored a primary TP53 N239S mutation in exon 7 accompanying with the EGFR L861Q mutation, which may contribute inferior clinical outcome of osimertinib.

There is little evidence regarding the effects of osimertinib in patients with EGFR mutations who also have concurrent mutations in RB1, TP53, and PTEN. Our present patient harboring concurrent mutations in L861Q, RB1 and TP53 received first-line osimertinib treatment and achieved PFS of 15.0 months. Osimertinib may be a therapeutic option for EGFR-mutant patients with concurrent mutations, and further investigations are required in this regard.

In conclusion, treatment of patients with uncommon mutations lacks established standard treatments. Our case shows that osimertinib demonstrated favorable activity in patients with NSCLC harboring concurrent uncommon EGFR, TP53, and RB1 mutations. In addition, dynamic ctDNA detection has implications for the development of treatment regimens, and the use of ctDNA as a biomarker is promising and will benefit both the clinic and patients.

## Data Availability Statement

The original contributions presented in the study are included in the article/supplementary material. Further inquiries can be directed to the corresponding authors.

## Author Contributions

All authors listed have made a substantial, direct, and intellectual contribution to the work and approved it for publication.

## Funding

This study was supported by funding from Science and Technology Program of Guangzhou China [grant number 202002030376], Guangdong Basic and Applied Basic Research Foundation [grant number 2021A1515011489] and Traditional Chinese medicine evidence-based capacity building project (grant number 2019XZZX --ZL001).

## Conflict of Interest

The authors declare that the research was conducted in the absence of any commercial or financial relationships that could be construed as a potential conflict of interest.

## Publisher’s Note

All claims expressed in this article are solely those of the authors and do not necessarily represent those of their affiliated organizations, or those of the publisher, the editors and the reviewers. Any product that may be evaluated in this article, or claim that may be made by its manufacturer, is not guaranteed or endorsed by the publisher.

## Abbreviations

EGFR-TKIs, Epidermal growth factor receptor tyrosine kinase inhibitors; NSCLC, non-small cell lung cancer; NGS, next-generation sequencing; AF, allelic fraction; PET-CT, positron emission tomography-computed tomography; FDG, fluoro-2-deoxy-d-glucose; PFS, progression-free survival; ctDNA, circulating tumor DNA; ORR, objective response rate.
